# The Ketogenic Diet for the Treatment of Mood Disorders in Comorbidity With Epilepsy in Children and Adolescents

**DOI:** 10.3389/fphar.2020.578396

**Published:** 2020-11-24

**Authors:** Francesca Felicia Operto, Sara Matricardi, Grazia Maria Giovanna Pastorino, Alberto Verrotti, Giangennaro Coppola

**Affiliations:** ^1^ Department of Medicine, Surgery, and Odontoiatry, Child and Adolescent Neuropsychiatry, University of Salerno, Salerno, Italy; ^2^ Department of Child Neuropsychiatry, Children’s Hospital “G. Salesi,” Ospedali Riuniti Ancona, Ancona, Italy; ^3^ Department of Pediatrics, University of Perugia, Perugia, Italy

**Keywords:** mood disorders, epilepsy, ketogenic diet, comorbidity, adolescents, young adults

## Abstract

The ketogenic diet, used for over a century as an alternative therapy for the control of drug-resistant seizures in both children and adults, has recently drawn increasing interest in various neurological or psychiatric disorders other than epilepsy. In particular, there are a few preliminary studies in mood and neurodevelopmental disorders such as anxiety, depression and autism spectrum disorders. Mood disorders in comorbidity with epilepsy are commonly seen in adolescents and young adults both at the onset and during the course of the epileptic disorder. The rationale for the use of the ketogenic diet is based on the potential mood stabilizing effects through level modifications of metabolites such as dopamine and serotonin and the regulation of GABA/glutamatergic neurotransmission, mitochondrial function and oxidative stress. In this review, epilepsies with a higher risk of mood disorders in adolescents will be considered. A brief overview of the various types of ketogenic diet that can currently be offered to young patients in order to improve palatability and compliance with the diet, is also included. The efficacy and tolerability of the ketogenic diet options for the treatment of mood disorders, with or without drug therapy including mood stabilizers and antidepressant drugs, are as well discussed.

## Introduction

Seizure disorders are the most frequent neurological pathology in developmental age, with 4–10% of children having at least one critical episode in this lifetime. Children with epilepsy also have a higher incidence of emotional and behavioral problems than their healthy peers. More specifically, disorders related to anxiety and depression are reported in adolescents in 15–36% and 8–35% of cases, respectively.

In clinical-based studies incidence is higher for both anxiety and depression (27–36% and 20–29%, respectively), compared to population-based studies (5% and 7–13%, respectively). On the other hand, psychiatric comorbidity in children with newly diagnosed epilepsy is significantly more frequent than in healthy controls, including not only anxiety and depressive disorders but also the attention deficit and hyperactivity/impulsivity disorder, the oppositional-provocative disorder, and tic disorders (Jones et al., 2007).

The same authors also report a higher recurrence of anxiety-depressive disorders in focal-onset compared to generalized epilepsies.

As for age, mood disorders are more frequently reported in adolescents than in pre-adolescents (Reilly et al., 2011) and symptoms are mainly represented by tearfulness, sadness, and becoming easily upset. Separation anxiety and social phobia are also frequent. In particular, the risk factors for depression in adolescents, are considered to be: epilepsy associated with a lower intelligence quotient, seizure recurrence, the syte of the epileptogenic focus and the type of antiseizure medications (Kwong et al., 2016).

These authors report that the internalizing problems are facilitated by the negative effect of the seizures in family members, by a poor emotional and communicative support to the family, by a maternal depressive symptomatology, by the negative psychological response to the disease and by the lack or loss of hope.

As for the relationship between seizure focus and depression/anxiety, Schraegle and Titus (2017) reported that psychopathological internalizing clinical symptoms were present in about 40% of 132 children and adolescents with generalized or focal epilepsy and that the group with temporal lobe epilepsy had more depressive symptoms than the group with frontal lobe epilepsy. The side of the epileptogenic focus was, however, not significant. In addition, the assessment of the health related quality of life found a link between parental depression and a higher number of antiseizure medications.

With respect to the relationship between anxiety/depressive symptoms and seizure frequency, Dehn et al. (2017) found that depressive symptoms and anxiety as well as the number of seizures decreased significantly from baseline to 6-months follow-up in patients with refractory epilepsy. In addition, the level of depression was a significant predictor of seizure frequency at 6-months follow-up. Depressive episodes can also be triggered by pharmacological changes according to three main modalities: after the introduction of an antiseizure medication with negative psychotropic properties, after the withdrawal of an antiseizure medication with stabilizing properties or after the introduction of an enzyme inducing antiseizure medication in patients receiving psychotropic drugs (e.g., antidepressants) (Barry et al., 2008).

A disorder of serotonin and norepinephrine neurotransmitters in the frontal and temporal lobes has also been reported as a potential risk factor of epilepsy and depression comorbidity (Kanner et al., 2012). In children with recent-onset epilepsy, an increased volume of the amygdala and a thinner cortex in the prefrontal and orbital region were found as well. The same dysfunctional networks have been described in anxiety disorders in the general population (Jones et al., 2015).

With respect to the treatment of mood disorders in children and adolescents with epilepsy, a psychoeducational and psychotherapeutic support to the patient and his/her family and a cognitive-behavioral approach should be mentioned, in addition to a correct diagnosis and optimization of seizure control., In more severe cases, a psychopharmacological therapy, including selective serotonin reuptake inhibitors and selective noradrenergic reuptake inhibitors, may be needed. Among the alternative non-pharmacological treatments, a ketogenic diet, used for over a century for the adjunctive treatment of epilepsies, is currently being evaluated in mood disorders (Coppola et al., 2019).

## Method

We conducted a comprehensive search of the PubMed, PsychINFO and Scopus electronic databases for peer-reviewed articles in the months of May to September 2020. The keywords were “depress*,” “manic depress*,” “bipolar disorder,” “mood disorder,” “anxiety,” “anxi*,” “psychiatry,” “mental disorder*” (Group 1) AND “ketogenic diet,” “ketosis,” “ketogenesis,” “ketone bodies,” “high fat low carbohydrate,” “diet,” “acetone,” “acetoacetic acid,” “beta-hydroxybutyric acid,” “acetylcoA,” “ketonemia,” “ketonuria,” “fatty acid metabolism,” “hyperketonemia,” “fasting,” “nutritional ketosis,” “acidotic” (Group 2). The keywords were combined as follows: Group 1 AND Group 2. Furthermore, a manual search for references of published articles was conducted.

## Ketogenic Diet (General Aspects)

The ketogenic diet is a diet rich in lipids and low in carbohydrates and protein, established as an effective non-pharmacologic treatment for many pathologies. It is a rigidly and individually calculated diet, strictly monitored in order to produce ketosis, thus simulating the metabolic changes during the starvation state (D’Andrea Meira et al., 2019).

The exact underlying mechanism of the ketogenic diet remains unclear. It is hypothesized to involve the following: alterations in mitochondrial function; inhibition of the mammalian target of rapamycin; effects of ketone bodies on neuronal function and neurotransmitter release, effects of fatty acids as antiepileptic and/or glucose stabilization. Ketone bodies may increase membrane potential causing hyperpolarization and *γ*-aminobutyric acid synthesis and, on the other hand, decrease the release of other neurotransmitters such as glutamate, norepinephrine, or adenosine (Masino and Rho, 2012).

Traditionally, the ketogenic diet has been considered for the treatment of metabolic diseases such as Glucose Transporter Protein 1 deficiency syndrome and Pyruvate dehydrogenase deficiency (Veggiotti et al., 2011). At present, the ketogenic diet has been reported as beneficial in several conditions such as infantile spasms, myoclonic-astatic epilepsy, and Dravet syndrome (Fejerman et al., 2005; Hong et al., 2010; Kossoff et al., 2018). Additionally, the ketogenic diet is an important alternative treatment for patients with refractory epilepsy for whom surgery was ruled out.

The classic ketogenic diet is calculated based on the ratio between fats and carbohydrates plus proteins. The most common ratio is 3:1 or 4:1, which means that 90% of the energy comes from fat and 10% from combined carbohydrates and proteins (Sampaio, 2016).

Over time, alternative ketogenic diet variants have been developed to make the treatment more liberal and palatable in order to increase patient’s compliance and to reduce adverse side effects.

The medium-chain triglyceride (MCT) diet was developed to make the ketogenic diet more palatable, allowing for a diet with a greater proportion of carbohydrate and protein. A randomized controlled trial comparing the MCT and classic versions of the ketogenic diet indicated there were no significant differences between the two diets. However, MCT oil is expensive and dietitians need to be trained to use MCT therapy (Neal et al., 2009).

The modified Atkins diet (MAD) has a ketogenic ratio of 0.9:1 (fat: carbohydrates and protein), with approximately 65% of the calories derived from fat sources. It is slightly more effective in those with a high seizure frequency at diet onset and in young adults (Kossoff et al., 2013).

The low glycemic index treatment (LGIT**)** is less restrictive than the classic ketogenic diet because it allows consumption of low glycemic index foods, while encouraging fat intake. The LGIT produces a lower level of ketosis than that of the classic ketogenic diet. The effect of the LGIT was shown to correlate with lower serum glucose levels at some time points (Coppola et al., 2011).

The choice of the diet protocol must be made on an individual basis considering the patient’s characteristics such as age, family circumstances, and severity and type of the illness to treat.

## Types of Epilepsy That Exhibit a Better Response to the Ketogenic Diet

Recent literature data and clinical experience in the real-world suggest that ketogenic diet can be effective in (some) patients with Dravet syndrome, in those with myoclonic-astatic epilepsy as well as in children with other forms of epilepsy and refractory epileptic syndromes, including refractory infantile spasms, Lennox-Gastaut syndrome, and seizures associated with tuberous sclerosis complex (Coppola et al., 2010; Veggiotti et al., 2011).

With regard to the topic of this review that is the ketogenic diet for the treatment of epilepsy and mood disorders, attention should be focused on the epilepsies that start or continue in the adolescent age, when mood disorders like anxiety, depression, mood instability and bipolar disorder, as well as anxious-phobic and obsessive behaviors, often occur in comorbidity with epilepsy (Jones et al., 2007; Coppola et al., 2019).

A ketogenic diet can be a suitable alternative treatment for refractory symptomatic focal-onset seizures with/without secondary generalization, awaiting for surgery or when a surgical solution has been excluded. A ketogenic diet can also be useful in patients with refractory primary generalized epilepsies.

In adolescents and young adults, ketogenic diet may be an effective additional treatment for both seizures and mood disorder. In fact, the ketogenic diet has been suggested to potentially act as a mood stabilizer (Arab et al., 2019). As to the relationship between age at diet onset and efficacy of the diet, the classic ketogenic diet is preferred in children under 2 years of age, while the MAD and the LGIT diet are preferred in adolescents and adults, because they are more suitable and increase patient’s compliance compared to the classic ketogenic diet (Kossoff et al., 2018; Miranda et al., 2012).

## Ketogenic Diet in Disorders Other Than Epilepsy

Over the past 10 years, there has been a growing interest in the use of the ketogenic diet in neurological and psychiatric disorders both in adults and in developmental age regardless of epilepsy. Neurological disorders in which ketogenic diet has been tried, are undoubtedly more numerous today, including metabolic disorders, brain trauma, headache, brain tumors, neurodegenerative disorders including Parkinson’s disease, Alzheimer’s disease, ALS and mitochondrial disorders as well as stroke. On the other hand, applications in the psychiatric field are more limited, including mainly anxiety, depression and autism spectrum disorders (Herbert and Buckley, 2013; Phelps et al., 2013; Verrotti et al., 2017; Wlodarczyk and Cubała, 2019).

## Ketogenic Diet and Mood Disorders

The mechanism of the ketogenic diet is complex and not fully clarified In fact, it involves metabolic changes in a variety of cellular signaling pathways, leading to decrease neuronal excitability and neuroprotection.

Bipolar disorders and other mood disorders are related to dysfunction of neurochemical balance in the brain, especially monoamines.

The beneficial effects of the ketogenic diet have been hypothesized in mood disorders, also based on the observational evidence that some antiseizure medications have mood stabilizing properties (eg, valproate, carbamazepine, lamotrigine) (El-Mallakh and Paskitti, 2001; Bialer, 2012).

The ketogenic diet was found to decrease the levels of metabolites of biogenic amines such as dopamine and serotonin which are neurotransmitters pivotal to the pathophysiology of depression (Dahlin et al., 2012). On the opposite, the ketogenic diet increased the levels of endogenous norepinephrine (Weinshenker, 2008). Furthermore, the ketogenic diet acts in multiple targets implicated in the pathophysiology of mood disorders. Beyond the regulation of neurotransmitters as GABA/glutamate transmission and monoamine levels, it has effects on mitochondrial function and biogenesis, neurotrophism, oxidative stress, insulin dysfunction, and inflammation (Brietzke et al., 2018).

So far, treatment of mood disorders first addressed changes in few targets, many of which linked to monoaminergic function, aiming at symptomatic control. Otherwise, the ketogenic diet, with its pleiotropic actions, may also have beneficial effects in the management of difficult-to-treat patients.

Preclinical studies, case reports, and case series have demonstrated mood stabilizing and antidepressant effects of the ketogenic diet (Murphy et al., 2004; Yankelevitch-Yahav et al., 2015; Brietzke et al., 2018; Grigolon et al., 2020).

Its use as adjunctive treatment in conjunction with pharmacotherapy has been found particularly effective in the management of resistant depression. In particular, the use of the ketogenic diet in the major depressive disorder has been reported to lead to improvements in both somatic and psychiatric symptoms, due to its modulation on GABAergic and glutamatergic levels. In this regard, although the etiology of depression is still largely unknown, GABA dysfunction has been implicated in the mood fluctuations in affective disorders (Lakhan and Vieira, 2008; Pehrson and Sanchez, 2015). Furthermore, the balance of GABA and glutamate neurotransmission regulates the neural activity, maintains proper brain function, and appropriate dopamine levels (Włodarczyk et al., 2017). Defects in the GABAergic neurotransmission in depressed patients may induce alterations in the dopaminergic concentrations. These alterations in the dopamine levels have been found to be related to depressive symptoms, like anhedonia (Wlodarczyk and Cubała, 2019). Ketosis by altering the GABA/glutamate ratio, could be a disease modifying treatment for major depression.

An energetic imbalance is known in mood disorders, due to changes in glucose, oxidative pathways, and mitochondrial functions. The ketogenic diet leads to changes in brain energy, which may be of benefit in global cerebral hypometabolism typical of the brains of depressed or bipolar patients. Moreover, the extracellular changes occurring during ketosis may decrease intracellular sodium concentrations, a common mechanism of action of effective mood stabilizers (El-Mallakh and Paskitti, 2001).

The potential efficacy of the ketogenic diet in the treatment of mood disorders was evaluated in animal models. Murphy et al. demonstrated that the rats on the ketogenic diet spent less time immobile, suggesting that rats on the ketogenic diet, like rats treated with antidepressants, are less likely to exhibit “behavioral despair.” This evidence suggests that the ketogenic diet acts like antidepressant drugs in the Porsolt swim test, a behavior despair test commonly applied in experimental studies to assess depressive-like behavior in rodents. Furthermore, the handling test revealed that the reduced immobility appeared to be due to the antidepressant properties of the ketogenic diet (Murphy et al., 2004; Yankelevitch-Yahav et al., 2015). The main literature data regarding the efficacy of the ketogenic diet in animal models and in humans are synthetized in Table 1.

**TABLE 1:** The ketogenic diet in mood disorders: preclinical and clinical studies.

Authors	Condition	Type of study	Sample size	Types of diet	Time of intervention	Main findings
Animal models						
Murphy et al. (2004)	Depression	Animal model (wistar rats)	20	4:1	7 days	Some evidence for potential antidepressant properties
Sussman et al. (2015)	Depression	Animal model (CD-1 mice)	20	4:1 *in utero*	30 days	Reduction of the susceptibility to anxiety and depression and increase hyperactivity
Ari et al. (2016)	Anxiety	Animal model (sprague-dawley and wistar albino glaxo/Rijswijk rats)	80	Ketone supplement	83 or 7 days	Reduced anxiety-related behavior
Guan et al. (2020)	Depression	Animal model (mice)	21	4:1	7 days	Improve in depressive-like behaviors
Humans						
Yaroslavsky et al. (2002)	Bipolar disorder	Case report human woman	1	4:1	1 month	No clinical improvement
Phelps et al. (2013)	Bipolar disorder	Case report human women	2	70% fat, 22% protein, 8% carbohydrate	2–3 years	Mood stabilization
IJff et al. (2016)	Depressive symptoms and cognition	Randomized-controlled trial - children and adolescents with drug -resistant epilepsy (1–18 years)	28 = KD group 22 = control group	4:1 = 2 pt, MCT = 20 pt, Only ketocal = 5 pt mixture = 1	4 months	Lower levels of anxious and depressive symptoms in KD group
Campbell and Campbell, (2019)	Bipolar disorder	Observational study of free-text comments on line	274	—	—	141 pt (85.5%) reported a positive impact of KD on mood stabilization
Zhang et al. (2019)	Depression quality of life	Single center quasi-experimental study children and adolescents with drug -resistant epilepsy (11–17 years)	42	LGIT (fat = 60%, protein = 25%, carbohydrates = 15%) moderate-intensity aerobic exercise	6 months	Improvement in depression levels and quality of life

KD, ketogenic diet; pt, patient; LGIT, low glicemic index diet; MCT, medium chain triglycerides.

Other experimental studies also revealed that ketogenic diet may have multiple beneficial effects, influencing cellular inflammatory response, preventing neurodegeneration, improving cognitive function, social and motor behavior, and anxiety symptoms (Grigolon et al., 2020).

In women affected by ovarian or endometrial cancer on ketogenic diet, Cohen et al. (2018) found an improvement in the perceived physical function status and energy, a reduction in hunger and craving for starchy food, and a natriuretic effect due to a decrease in insulin levels and increase in ketone bodies (Cohen et al., 2018), overall leading to an improvement of the quality of life in these patients.

McClernon et al. (2007), examined the effect of diet on mood, hunger, and other associated symptoms in obese patients, revealing hunger reduction and lower food cravings and a reduced negative affect in ketogenic diet-fed patients compared with those on a low-fat diet. The Authors suggested that the favorable effects on hunger could be explained by the intake of only one food class. Moreover, the stabilization of blood glucose levels due to the ketogenic diet could reduce food craving and improve energy levels. The reduction of negative affect confirmed the antidepressant and mood stabilizing properties of the ketogenic diet previously reported in experimental studies on animal models.

Other studies, particularly in obese patients, highlighted an improvement in physical functioning and mental health in those on ketogenic diet, a greater perceived control, and a lower level of depression, leading to an overall improvement in the quality of life also in the long-term. The ketogenic diet improved as well glycemic control associated with smaller fluctuations in glucose and insulin serum levels, which may improve vitality and mood (Foster et al., 2003; Samaha et al., 2003; Yancy et al., 2009).

Many observational studies on epileptic patients receiving ketogenic diet reported an improvement in their behavior and cognitive functions, in terms of better attention skills and alertness, level of activity, socialization, and better quality of sleep (Nordli et al., 2001; Pulsifer et al., 2001; Hallböök et al., 2007; Lambrechts et al., 2013; Chianese et al., 2018). Recently, IJff et al. (2016) demonstrated a positive impact of the ketogenic diet on behavior and cognitive functioning in children and adolescents affected by refractory epilepsy, which was independent of the improvement of seizure control. In particular, they found an improved activation, less anxiety and mood disturbed behavior, increased productivity, increased mental alertness and improved attention, and cognitive activation in patients on ketogenic diet compared with those on usual standard care. Similarly, Lambrechts et al. (2012) found a substantial improvement in mood and better quality of life in adult patients with chronic refractory epilepsy treated with ketogenic diet.

All these sparse evidences highlight the favorable impact of the ketogenic diet on brain processes underlying mood, behavior, and cognition, emphasizing by consequence its therapeutic efficacy for emotional regulation and improvement, maladaptive and disturbed behavior, and other mood disturbances.

## Perspectives and Conclusion

The prescription of the ketogenic diet therapy increased rapidly worldwide with a broader understanding of its benefits for epilepsy and other disorders. Less restrictive versions of the diet have been developed to meet the needs of adolescents and adults, and increase their compliance.

Because of its well-defined multi-systemic beneficial effects, including also neurotrophic, antioxidant, neuroprotective, and anti-inflammatory properties in the central nervous system, the ketogenic diet acts in critical targets for illnesses trajectory.

Furthermore, its efficacy in seizure management in the setting of modern neuropharmacology makes the ketogenic diet remarkable. Its prescription has been widened to other applications, providing a rationale for repurposing this therapy for other brain-related disorders. Due to its potential pleiotropic benefits, the ketogenic diet should be considered as a promising intervention in research in mood disorders, particularly in the treatment of drug resistant cases.

In most cases, the symptomatic approach of treatment of brain dysfunction may suggest that science could move toward the functional dysregulation of brain metabolism, mitochondrial homeostasis, and synaptic plasticity. In this regard, the “metabolic therapy” of the ketogenic diet could be one of the most promising interventions.

Although definitive clinical evidence for expanded use of the ketogenic diet outside of epilepsy is still scanty, there are ongoing studies for a few neurological conditions assessing its efficacy on improving symptoms and even the disease processes themselves. Most of the experimental evidence has been focused on the concept that the broad efficacy of the ketogenic diet may be due mainly to the normalization of energy metabolism dysregulation (Masino and Rho, 2012). The notion that other neurological disorders beyond epilepsy might be linked in their pathophysiology to energy imbalance provides a rationale for how the ketogenic diet may improve an underlying dysfunction and even may prevent and revert symptoms (Pathak et al., 2013).

The type of the ketogenic diet for the treatment of mood disorders is not specified in two reports (IJff et al., 2016; Campbell and Campbell, 2019). In another study (Zhang et al., 2019), the effect on quality of life of a low glicemic index diet, together with a home-structured exercise therapy, was assessed in children with epilepsy. Combined therapy demonstrated a promising improvement in depression level and quality of life.

Besides the growing body of evidence, further clinical and translational research on the effectiveness of the ketogenic diet and its mechanisms of action, might increase its prescription in the management of epilepsy and other neuropsychiatric disorders, not only in difficult to treat cases but also as an emerging therapy in resource-constrained as well as developed societies worldwide.

## Author Contributions

FO and SM equally contributed to the manuscript. GP searched for literature data. AV and GC programmed and supervised the review shedule.

## Conflict of Interest

The authors declare that the research was conducted in the absence of any commercial or financial relationships that could be construed as a potential conflict of interest.
